# Rapid personalized chemotherapy recommendation for cancer patients: An interview with Shaohua Ma and two members of his group

**DOI:** 10.1016/j.patter.2021.100387

**Published:** 2021-11-12

**Authors:** J.Q. Fan, Y.L. Feng, S.H. Ma

**Affiliations:** 1 Tsinghua University, Shenzhen International Graduate School (SIGS), Shenzhen 518055, China; 2 Tsinghua-Berkeley Shenzhen Institute (TBSI), Shenzhen 518055, China

## Abstract

Shaohua Ma, an early-career group leader, and his team talk about their passion for data science and their project published in *Patterns*, where multiplex gene quantification-based “digital markers” are used for extremely rapid evaluation of chemo-drug sensitivity. This method allows quick and personalized chemo-drug recommendations for cancer patients, helping to improve their clinical care and health outcomes.

## Main text

### Shaohua, Jiaqi, and Yilin, please tell us a little about yourselves and your recent paper published in *Patterns*

**S.H.Ma:** My research focuses on organoid engineering, intelligent microfluidics, and computational biology and medicine, for personalized and regenerative therapy.

My team is based in Shenzhen, China, a young city known for high-end innovation. I started here in late 2017. In 2018, the first group of students joined my lab, and now there are over 20 students. Data science in biomedicine is developing fast and benefits from the contributions of both the computational community and the biomedical community. The latter group contributes by intentionally harvesting high-quantity and high-quality data for information processing and extraction. Further, databanks are established to facilitate the spread of computational power in these fields. In my opinion, data science has a supreme convergence opportunity with various biomedical disciplines, with the expected scope far beyond the current level. Understanding networks and probing critical cues are lightening the path for therapeutic exploration. In our recent work published in *Patterns*,^1^ we propose “digital markers” for choosing personalized cancer medicine. We will work further on this concept until it satisfies clinical use. We decided to publish this work in *Patterns* because it is an excellent platform for displaying and sharing novel ideas.

**J.Q.Fan:** I am a third-year PhD student majoring in data science and information technology. I have studied in the same field as an undergraduate student. With the fast development of diverse data collection technologies in all kinds of research fields, people can obtain abundant data, which helps us to uncover many mysteries of science. This gives a great chance and challenge to data researchers. Whether data researchers can delve for useful information from a large amount of data is the key to further research. This attracts me a lot and guides me on this path. In my opinion, data science is the science about data organization and information acquisition. Data scientist refers to one who can choose and develop appropriate approaches to organize and analyze data in diverse situations. I think this needs enough knowledge, inspiration, and experience. I don’t define myself as a data scientist yet because I am not experienced enough, but I aim to be one. We decided to publish in *Patterns* because it is a great and open-minded journal that concerns data science and relative interdisciplinary researches, which we believe is a wonderful platform to present our work.

**Y.L.Feng:** I am a third-year PhD student at Tsinghua University. I major in precision medicine and healthcare research. I am doing some interdisciplinary research that includes data science, neuroscience, and biomedicine. As a biomedical student, I believe that data science helps solve problems in the clinical field. Given the development of technology and science, the big data of bio-information need to be cleaned and analyzed using data science solutions. In our research field, we always use algorithms to search correlations in proteomic data, genomics data, and so on. *Patterns* is not only a journal for data scientists but also a journal for interdisciplinary researchers, and that is why we chose *Patterns* for the publication of our paper.

### Shaohua, tell us about the research in your team, what drew you to this area of research? How has the research focus of your team evolved over the years?

**Figure 1:**
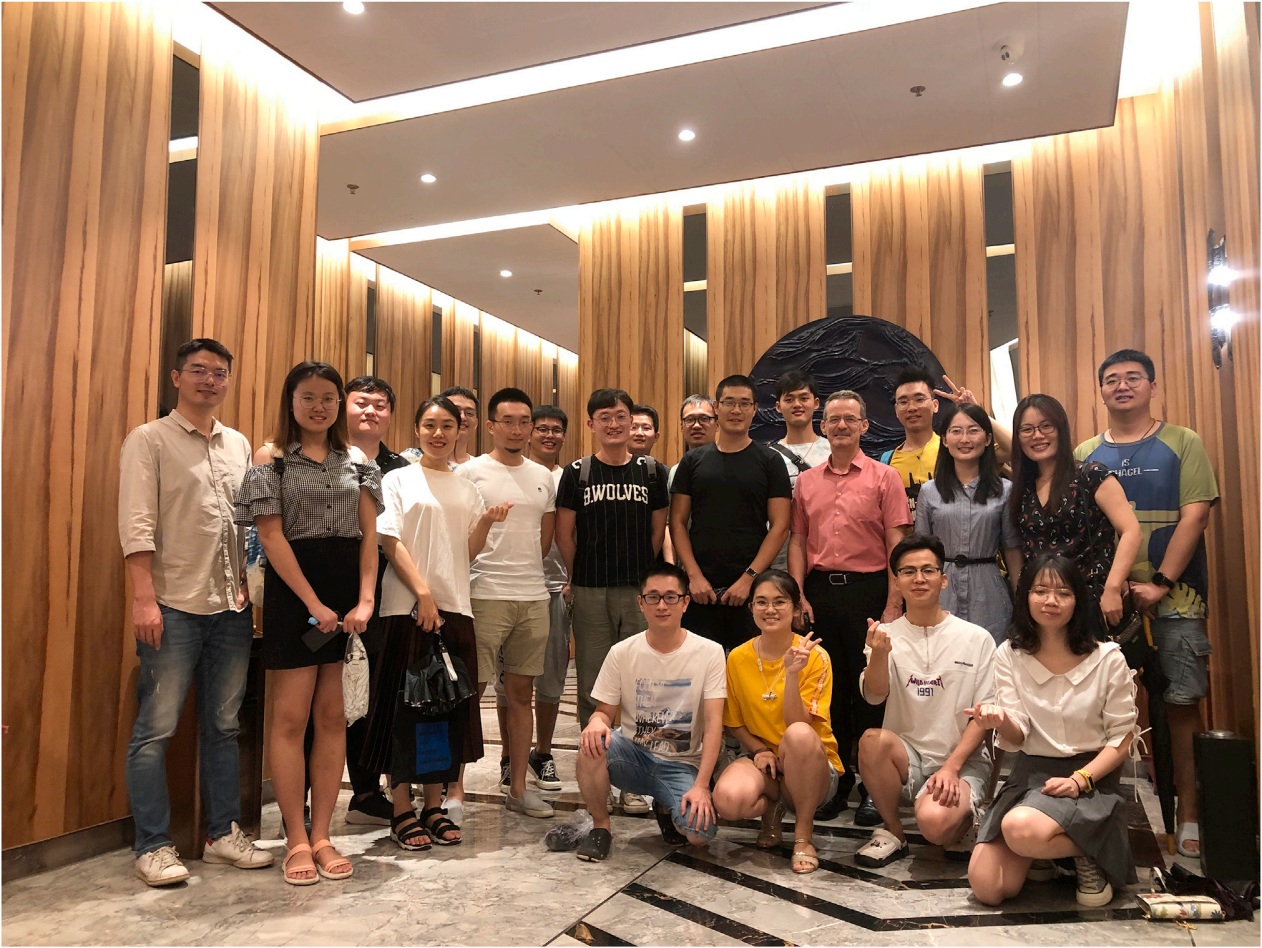
Group photo of S.H. Ma's Lab

**SHM:** My team is a multi-disciplinary team (Figure 1). The students are from different backgrounds in biomedicine, mechanical and electronic engineering, and data science. We converge biomedicine and engineering to achieve organoid engineering goals including design, production, and therapeutic uses of organoids possessing patients’ tumor properties. We also converge biomedicine and data science to explore regulatory cues in disease and indictive cues in therapy. Moreover, we combine engineering and data science to find representative cues to evaluate interference (e.g., mechanical processing of living samples) and setup manufacturing standards. A project starts with reading motivations from industry and clinics, decoding these motivations into laboratory goals, and eventually wrapping up in extra-laboratory practices.

Our ultimate aim is to bridge laboratory research and technology translation.

### Shaohua, how did this project come to be?

**SHM:** Chemotherapy for cancer patients has long been a tough nut to crack. It depends on the experience and ability of the doctors and follows arbitrary standards. As a result, the prognosis is often poor and unpredictable. Some published works used gene expression profiles to predict cancer drug responses. But these works cared more about the prediction accuracy than timely efficacy, which is more important in real practices. A patient cannot wait for over a month for a “suggestion.” Rapid drug recommendation is highly demanded. So we studied to establish a set of digital markers that hopefully can help to predict drug response and give personalized drug recommendations in an extremely short time.

### Yilin, what drew you to your current team and topic?

**YLF:** I worked as an intern in a hospital before. With the high demand for timely diagnosis and treatment, doctors should collaborate with data scientists in order to meet the demand. That’s why I came to this topic and team.

### Jiaqi, as the first author of this paper looking back, what advice would you have given yourself at the start of the project? Is there anything you would have done differently?

**JQF:** We have iteratively updated our ideas and methods many times during the whole process, and we have also overcome many difficulties both in the computational approach and wet-lab experiments. In the beginning, we spent a lot of time analyzing cancer cell lines and struggled with the limited data size. Looking back, if I had another chance, I would focus on cancer cell lines and patient samples at the same time and carry out wet-lab experiments earlier. I would also learn more about gene analysis at the start of the project.

### Jiaqi and Yilin, what’s next for the project? And what’s next for you?

**JQF:** For the project, we will update the computational model and improve the performance of our digital marker-based drug recommendation method; on the other hand, we will transfer our research into society and industry.

For me, I will still focus on the interdisciplinary research on data science and biomedicine. Apart from cancer digital markers, I am also interested in neuroscience and neuroimaging.

**YLF:** I think the application in a clinical trial will be the next step for the project since we want to transfer our research into society and industry. I will focus on the next step and work more on this project

### Yilin, what is the role of data science in the domain/field that you work in? What advancements do you expect in data science in this field over the next 2–3 years?

**YLF:** In our field, there is a subject named biomedical data science that focuses on the creation of novel methodologies to advance biomedical science discovery. This subject includes collecting big data of bio-information and annotating, cleaning, organizing, storing, and analyzing them to extract knowledge. Over the next couple of years, I expect data science will become an indispensable role in our field.

### Shaohua and Jiaqi, how do you keep updated with both advances in data science techniques and the advances in the field/domain that you work in?

**SHM:** I keep updated with advances by reading the latest papers, organizing journal clubs in my lab, attending forums and conferences, and communicating with community peers. Communication is a great way to learn quickly and thoughtfully, especially something not in my original field. Most importantly, TBSI and SIGS are the places designed in the first place to conduct transdisciplinary research and education.

**JQF:** I usually keep myself updated with the advances by browsing the latest news and published papers, listening to talks, and communicating with others. For the topics and methods that interest me best, I read carefully and go through the supplementary materials and even the codes.

### Jiaqi, tell us about any barriers you faced in pursuing data science as a career

**JQF:** Working on data science, especially data science in interdisciplinary research, is not easy. The first barrier for me is to grasp variable classical methodologies and keep updated with the latest advances. To apply the methods to solve the problems, I need to understand them well. The second barrier is to look deep into the problems that are not familiar to me previously and connect them to the approaches in data science. For example, in this project, I need to understand gene expression, cancer, and drug treatment and think about data-science-based drug recommendation method.

### Jiaqi, what attributes make a data scientist successful?

**JQF:** In my opinion, a successful data scientist or one who aims to be a successful data scientist should have a solid foundation in mathematics, a good sense of the new advances, and a deep understanding of the problems and the field/domain in which they work.

“Practice makes perfect.” I believe nothing happens of a sudden. Working hard and gathering experience are the foundations for everything.

### Yilin, what is the fun part of being a data scientist?

**YLF:** Being a data scientist should be skilled on mathematical modeling, compared to data analysts, which I think is the fun and also difficult part. And data analyst is just the first step of being a data scientist.

### Shaohua, which of the current trends in data science seem the most interesting to you? In your opinion, what are the most pressing questions for the data science community?

**SHM:** The progression of artificial intelligence interests me most, especially small-sample learning, self-supervised learning, and interpretability of models, which may fit any challenging problems in biomedical research. In my opinion, the interpretability of artificial intelligence and the correct use of statistical methods are the most pressing questions for the data science community.

### Shaohua, what is your advice for future data scientists?

**SHM:** First, a good sense of data is fundamental. Second, keep an open mind to new advances in data science and important problems in other research fields. Third, gather enough experience in coding and solving problems.
